# EEG functional connectivity metrics wPLI and wSMI account for distinct types of brain functional interactions

**DOI:** 10.1038/s41598-019-45289-7

**Published:** 2019-06-20

**Authors:** Laura Sophie Imperatori, Monica Betta, Luca Cecchetti, Andrés Canales-Johnson, Emiliano Ricciardi, Francesca Siclari, Pietro Pietrini, Srivas Chennu, Giulio Bernardi

**Affiliations:** 1 MoMiLab Research Unit, IMT School for Advanced Studies Lucca, Lucca, Italy; 2 Department of Psychology, University of Cambridge, Cambridge, United Kingdom; 3 Center for Investigation and Research on Sleep, Lausanne University Hospital, Lausanne, Switzerland; 4 School of Computing, University of Kent, Chatham Maritime, United Kingdom; 5 Department of Clinical Neurosciences, University of Cambridge, Cambridge, United Kingdom; 6 University Hospital of Pisa, Pisa, Italy; 7 Center for Social and Cognitive Neuroscience (CSCN), School of Psychology, Universidad Adolfo Ibáñez, Santiago, Chile; 8 The Neuropsychology and Cognitive Neurosciences Research Center (CINPSI Neurocog), Universidad Católica del Maule, Talca, Chile

**Keywords:** Dynamical systems, Biophysical models

## Abstract

The weighted Phase Lag Index (wPLI) and the weighted Symbolic Mutual Information (wSMI) represent two robust and widely used methods for MEG/EEG functional connectivity estimation. Interestingly, both methods have been shown to detect relative alterations of brain functional connectivity in conditions associated with changes in the level of consciousness, such as following severe brain injury or under anaesthesia. Despite these promising findings, it was unclear whether wPLI and wSMI may account for distinct or similar types of functional interactions. Using simulated high-density (hd-)EEG data, we demonstrate that, while wPLI has high sensitivity for couplings presenting a mixture of linear and nonlinear interdependencies, only wSMI can detect purely nonlinear interaction dynamics. Moreover, we evaluated the potential impact of these differences on real experimental data by computing wPLI and wSMI connectivity in hd-EEG recordings of 12 healthy adults during wakefulness and deep (N3-)sleep, characterised by different levels of consciousness. In line with the simulation-based findings, this analysis revealed that both methods have different sensitivity for changes in brain connectivity across the two vigilance states. Our results indicate that the conjoint use of wPLI and wSMI may represent a powerful tool to study the functional bases of consciousness in physiological and pathological conditions.

## Introduction

Functional connectivity (FC) metrics identify statistical (undirected) associations among spatially distinct brain areas. Electroencephalography (EEG) and magnetoencephalography (MEG) represent popular neuroimaging modalities for the estimation of FC owing to their high temporal resolution, in the order of milliseconds. However, both EEG and MEG suffer from volume conduction, which results from the instantaneous propagation of electric fields generated by a primary current source to all (or most) of the on-scalp sensors. Because of this linear mixing of different sources on the same sensor, common methods for FC estimation, such as coherence or mutual information, may lead to the identification of apparent functional couplings that do not reflect true brain inter-regional interactions^1–3^. To overcome this problem, several new FC methods have been specifically designed to minimize the impact of volume conduction effects. In particular, the weighted Phase Lag Index (wPLI^1^) and the weighted Symbolic Mutual Information (wSMI^4^), represent examples of spectral (wPLI) and information-theoretic (wSMI) connectivity estimation methods that are increasingly applied to both EEG and MEG data^5–19^. These two connectivity metrics are modified versions of pre-existing methods (PLI^1,20^; SMI^4^) that minimise the contribution of ‘(almost-)zero-lag’ interactions, potentially determined by volume conduction. These approaches are thus expected to allow identifying true time-lagged functional couplings^21–25^ in the activity of underlying brain sources, while excluding apparent zero-lag connectivity driven by a mixture of real and spurious relationships^26,27^.

Both wPLI and wSMI have been applied to explore brain functional dynamics associated with different behavioural states^6,12^ or potential network-level alterations in pathological conditions (e.g., Alzheimer’s disease^13^, multiple sclerosis^14^, schizophrenia^15^ and social anxiety disorder^16^). Interestingly, they have also been suggested to allow the identification of variations in functional integration accompanying changes in the level of consciousness^4,8–10,17–19^. For instance, King and colleagues^4^ found that wSMI connectivity between centro-posterior areas and other brain regions is higher in healthy conscious individuals as compared to patients with *unresponsive wakefulness syndrome* (UWS) or in a *minimally conscious state* (MCS). Similarly, Chennu and colleagues^17,19^ showed that alpha-band wPLI-based functional networks differ between healthy individuals and patients with disorders of consciousness (UWS, MCS). In line with this, previous studies^9,18^ also showed that propofol sedation in healthy individuals is associated with a decrease in alpha-band wPLI^18^ and a relative increase in delta-band wPLI connectivity^9^. These observations across different conditions characterised by altered levels of consciousness are particularly interesting, as they suggest that wPLI and wSMI may offer general, relatively simple and reproducible indices of the current level of consciousness of an individual^28^.

In spite of these promising findings, it is currently unclear whether the two methods provide a similar description of brain inter-regional relationships or account instead for distinct types of functional interactions. In fact, wPLI^1^ is a measure of phase synchronisation that may account for linear interactions but is also expected to be sensitive to nonlinear couplings^29,30^. On the contrary, wSMI^4^ is thought to reveal nonlinear relationships due to its grounding in information theory^31^. However, the actual performance of the two methods at detecting distinct types of connectivity dynamics has never been directly compared in simulated or real experimental data. Therefore, here we used simulated high-density (hd-)EEG data to specifically investigate and compare the accuracy of wPLI and wSMI in identifying different types of interaction dynamics, including both linear and nonlinear dependencies. In addition, to evaluate the potential impact of differences between the two methods on the analysis of real experimental data, we tested wPLI and wSMI on hd-EEG recordings collected from human participants in distinct behavioural states, namely wakefulness and deep (N3-)sleep, typically characterised by markedly different levels of consciousness^32^. In light of previous observations suggesting that the two methods may allow the detection of differences in the level of consciousness^4,7–10,17–19^ we expected both wPLI and wSMI connectivity to differ between wakefulness and N3-sleep. However, here we also asked whether the two connectivity metrics provide overlapping or complementary information about changes in brain functional dynamics across the two vigilance states.

## Results

### Simulation of linear and nonlinear interdependencies in hd-EEG data

**Figure 1:**
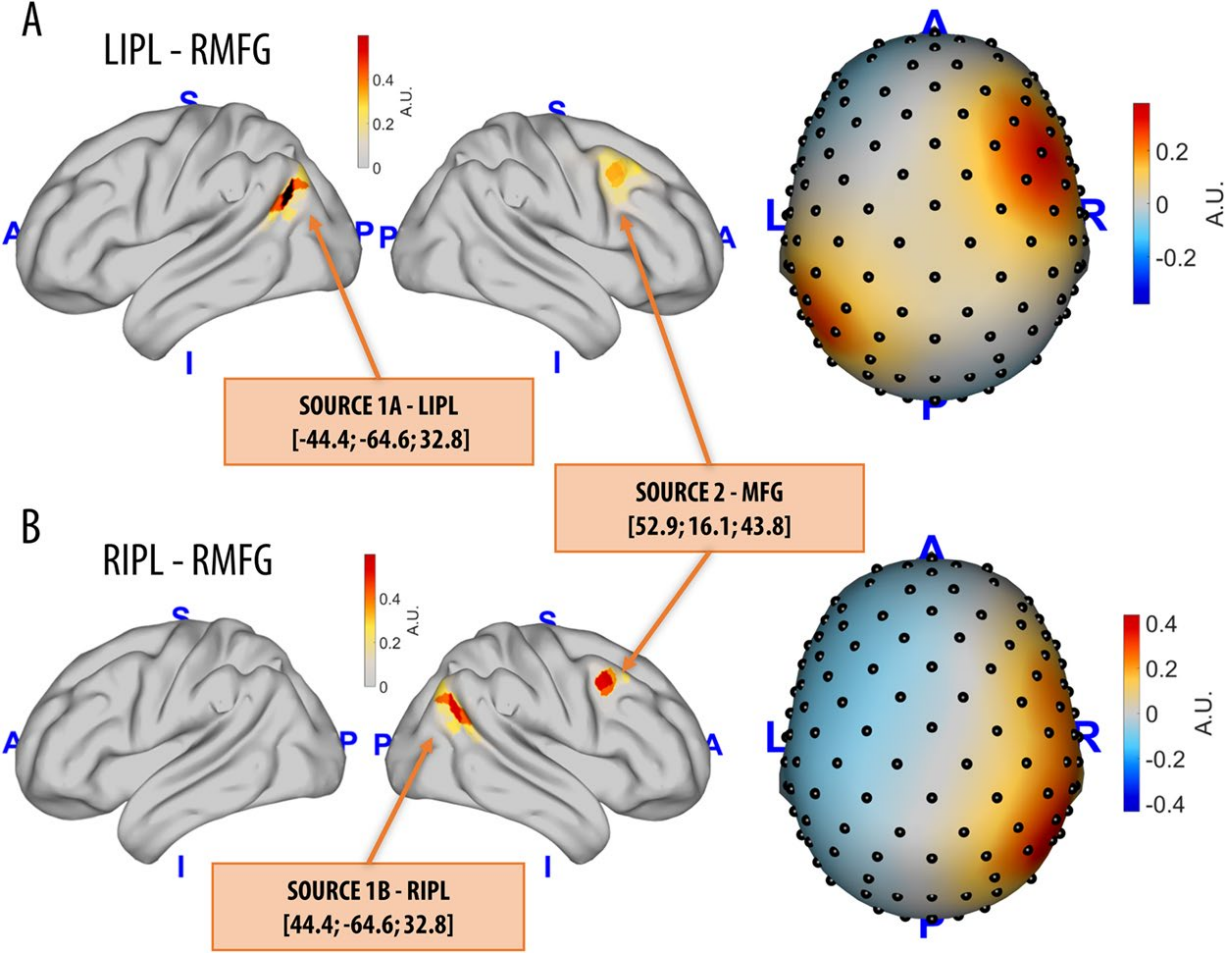
From modelling source dynamics to EEG field patterns. Intra- and inter-hemispheric interactions between two source pairs were modelled: the first source was placed either in left (LIPL) or right (RIPL) inferior parietal lobule, while the second source was kept in the right middle frontal gyrus (RMFG). Source amplitudes are shown using a lateral view of the brain, while resulting EEG field potentials are plotted using a top view of the scalp (A.U. stands for arbitrary unit). The brain images were plotted using the Matlab functions provided in the BBCB toolbox^33^.

**Figure 2:**
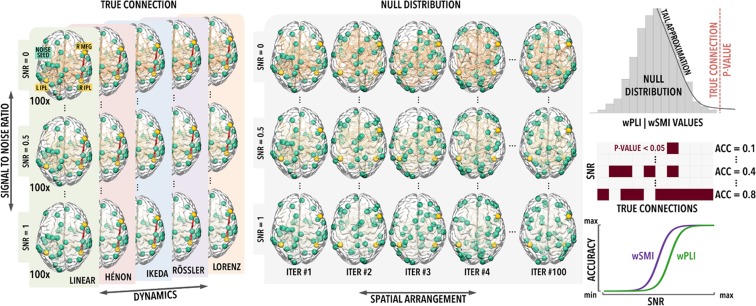
Outline of the methodological design for the assessment of whole-brain accuracy. The source locations LIPL, RIPL and RMFG are marked as yellow dots in the brain plots, while the red line indicates a true interaction between two of the sources (RIPL-RMFG). For each SNR in the range 0.01–1.00 (0.01 steps; N = 100) different spatial distributions (N = 100) of random background noise (marked as green dots) were generated in combination with true interactions between the source pairs and projected at scalp level. The corresponding null distributions were obtained through time-point-shuffling of the original interacting source-level timeseries. The same procedure has been applied to all interaction dynamics and tested source pairs (intra/inter-hemispheric). The brain images were rendered using Surf Ice (vers. 5 May 2016, 64-bit).

The MATLAB-based (The MathWorks, Inc., Natick, Massachusetts, USA) ‘Berlin Brain Connectivity Benchmark’ (BBCB) framework^33^ was used to simulate scalp-level hd-EEG recordings (108 channels, 500 Hz, 120 s) including bivariate relationships between two cortical sources. We modelled an intra-hemispheric interaction, between the right inferior parietal lobule (RIPL) and the right middle frontal gyrus (RMFG), and an inter-hemispheric interaction, between the left inferior parietal lobule (LIPL) and the right middle frontal gyrus (RMFG) (Fig. 1). The choice of these locations was motivated by previous neuroimaging studies showing that resting-state activity of these areas is modulated by conscious perception and attention^34–37^. As detailed in the Materials and Methods section, we simulated nine different coupling relationships between the two sources, respectively based on linear autoregressive (AR) model, Hénon map, Ikeda map, Rössler (x, y), Rössler (x, z), Rössler (y, z), Lorenz (x, y), Lorenz (x, z) and Lorenz (y, z) (see below for details). For each pair of source locations (LIPL-RMFG and RIPL-RMFG) and each type of simulated source coupling dynamics we modelled 100 different signal-to-noise ratios (SNR; from 0.01 to 1, with steps of 0.01), which describe the weighting of simulated source signals with respect to simulated background activity. Moreover, 100 different background noise patterns were obtained for each considered SNR. As detailed below, the accuracy of wPLI and wSMI at detecting the different interaction dynamics was thus computed both across patterns of noise distribution (for accuracy at each SNR) and across SNRs (for an estimate of overall accuracy) (Fig. 2).

**Figure 3:**
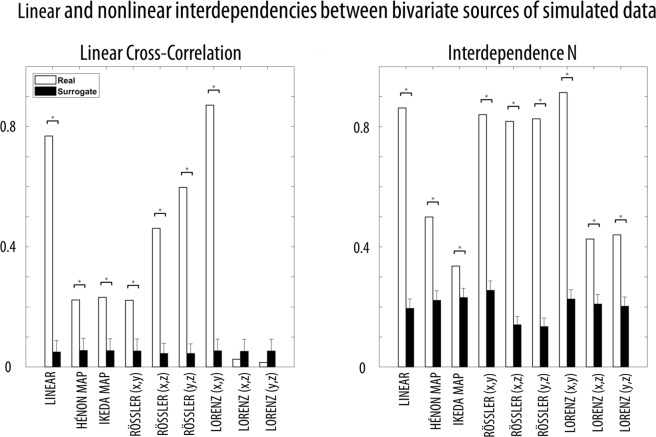
Linear and non-linear interdependencies between bivariate sources of simulated data. The absolute value of cross-correlation (CC; measure of similarity of two series as a function of the displacement of one relative to the other) and the interdependence measure N (measure of the nonlinear relationship between two time series) were computed for simulated true source time-series (0.5–12 Hz) and the null distribution, obtained by shuflling the source time-series (N = 1000, 0.5–12 Hz). For both CC and N, low values indicate independence, while high values indicate strong dependence. The differences between the true simulated data and its null distribution, i.e. surrogate data, were computed (* for p_one-tail_ < 0.05, Bonferroni-corrected based on 18 comparisons). The error bars show the standard deviation for the null distribution. For all cases, N was computed using the following parameters (but very similar results were obtained when optimal, individual parameters were selected for each time-series): embedding dimension (m = 10), time lag (tau = 5), theiler correction (theiler = 50), number of nearest neighbours (nn = 10).

First, we quantified the content of linear and nonlinear interdependencies in the nine examined interaction dynamics. In particular, to quantify the linear content of the bivariate relationships between the original sources we used cross-correlation, which offers a simple measure of similarity of two signals as a function of the displacement of one relative to the other^38^. In order to measure the nonlinear content, we took the average of the directional, nonlinear interdependence measure N in both directions of the source dynamics^38,39^. Most of the modelled interaction dynamics presented a mixture of linear and nonlinear dependencies, with the notable exception of Lorenz (x, z) and Lorenz (y, z), which showed a clear predominance of nonlinear interactions (Fig. 3).

### Simulated data - whole-brain connectivity

**Figure 4:**
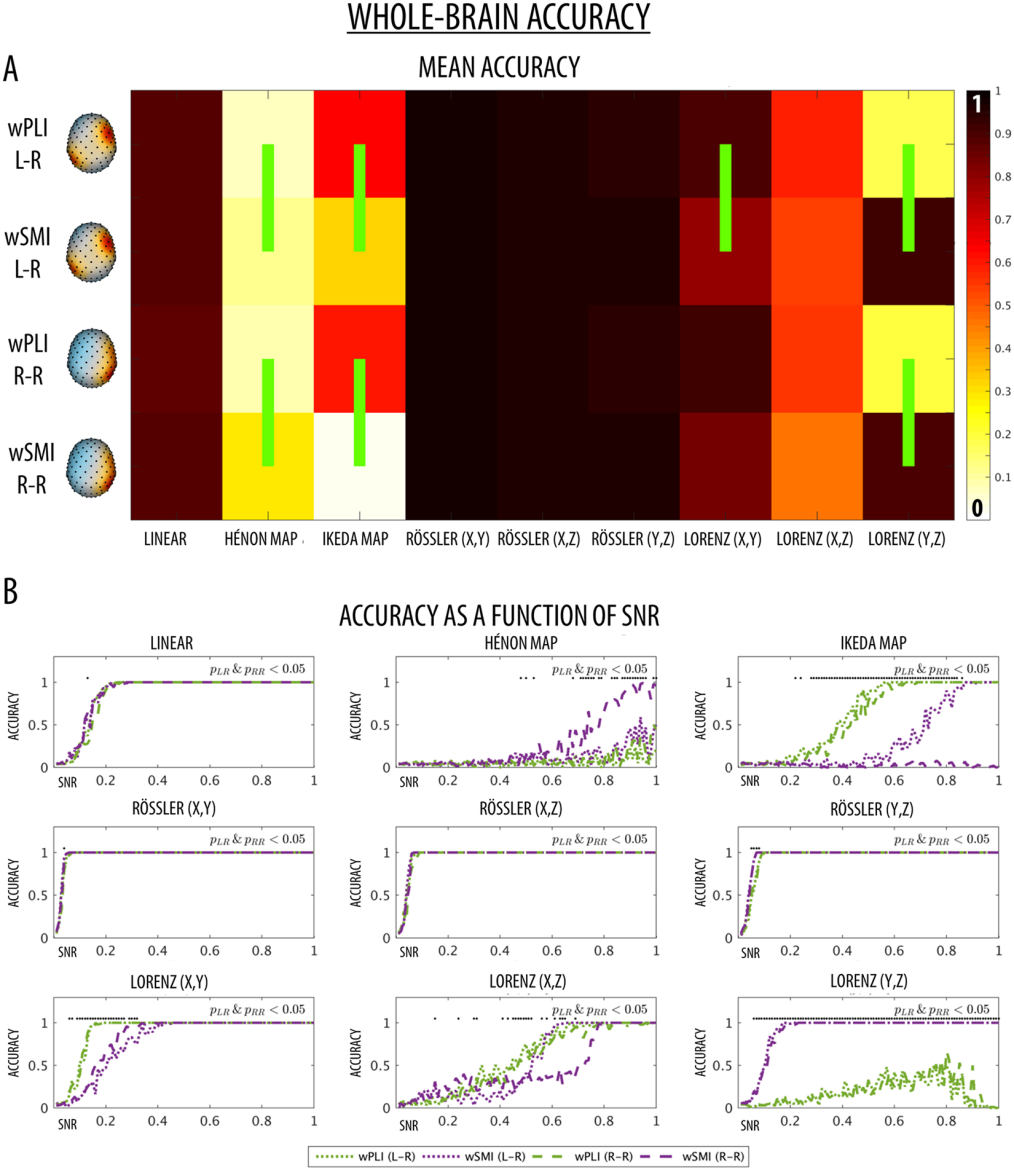
(**A**) Mean whole-brain detection accuracy for all nine different relationships between the chosen source location pairings (L-R = left IPL to right MFG; R-R = right IPL to right MFG). The green vertical lines mark significant differences between wPLI and wSMI (permutation tests, p < 0.05) for each type of interaction, pairing of source locations and SNR. The brain images were plotted using the Matlab functions provided in the BBCB toolbox^33^. (**B**) Whole-brain detection accuracy for all nine different relationships between the chosen source location pairings as a function of SNRs. Black dots at the top of each graph mark significant accuracy differences between wPLI and wSMI for each specific SNR that were observed for both intra- and inter-hemispheric conditions.

The whole-brain detection accuracy was computed as the proportion of cases in which the whole-brain median connectivity value (across all channel-pairs) of each simulated EEG dataset passed the 95th percentile of the corresponding null distribution. The null distribution consisted of whole-brain median connectivity values that were computed in matched simulated EEG datasets, where the time series between cortical sources of interest were subjected to one of two different surrogate procedures (time-point-shuffling or AAFT-randomization) to destroy their interaction relationship (see Figs 1 and 2). Figure 4A shows the mean accuracy of wPLI and wSMI (averaged over all SNRs) computed for each source pairing (intra/inter-hemispheric) and tested interaction dynamics. Figure 4B shows the whole-brain accuracy at each SNR. Of note, the accuracy of the two connectivity measures was similar for intra- and inter-hemispheric connections. The performance of both metrics was similar for the linear relationship in the broadband (0.5–12 Hz) signal. However, wPLI showed higher accuracy than wSMI in the intra-hemispheric case when connectivity in the alpha-band (8–12 Hz; corresponding to the range in which the interaction was modelled), was specifically considered (Fig. S1). While wSMI performed better at detecting the Hénon map dynamics for high SNRs (≥0.67), wPLI performed better at detecting the Ikeda dynamics, especially at intermediate SNRs (0.28–0–86). Both wPLI and wSMI showed significant and comparable levels of accuracy for all Rössler (x, y; x, z; y, z) cases at all tested SNRs, with the exception of low SNRs (Rössler (y, z) SNRs 0.05–0.08), for which wSMI tended to achieve a better detection performance. For the Lorenz (x, y) dynamics, wPLI achieved a better mean intra-hemispheric accuracy relative to wSMI, with the strongest differences observed for low SNRs (0.06–0.32). On the other hand, wSMI had higher accuracy for identifying Lorenz (y, z) dynamics for all SNRs ≥0.06. Finally, while no overall performance differences were observed at detecting Lorenz (x, z)-based interaction dynamics, wPLI tended to achieve a higher accuracy for intermediate SNRs, between 0.41 and 0.51. Of note, with the expected exception of the Rössler dynamics (see Methods), similar results were obtained when the null distributions were generated using phase-shuffling (AAFT) instead of time-point shuffling (Fig. S2).

### Simulated data - topographic connectivity

**Figure 5:**
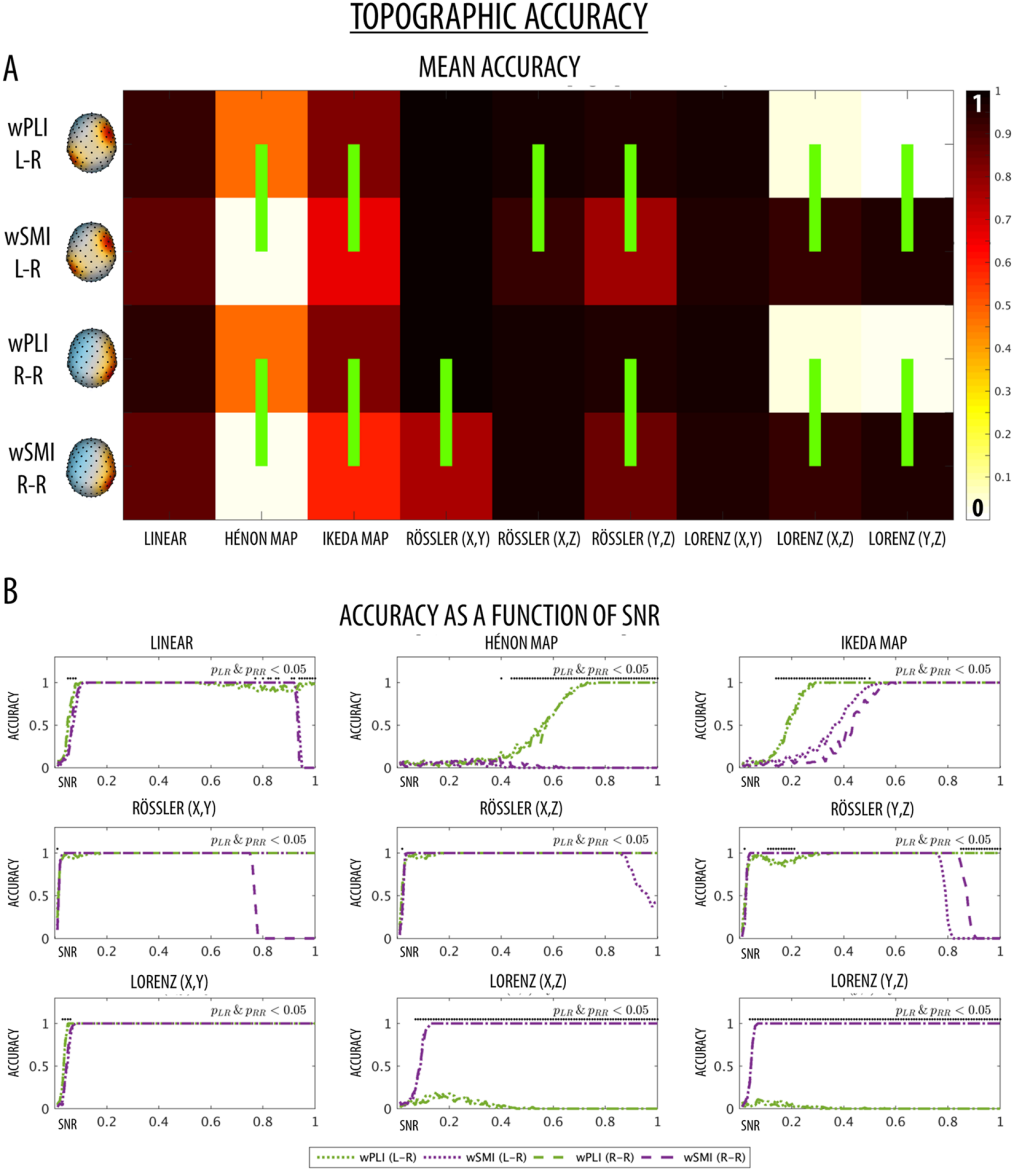
(**A**) Mean topographic detection accuracy for all nine different relationships between the chosen source location pairings (L-R = left IPL to right MFG; R-R = right IPL to right MFG). The green vertical lines mark significant differences between wPLI and wSMI (permutation tests, p < 0.05) for each type of interaction, pairing of source locations and SNR. The brain images were plotted using the Matlab functions provided in the BBCB toolbox^33^. (**B**) Topographic detection accuracy for all nine different relationships between the chosen source location pairings as a function of SNRs. Black dots at the top of each graph mark significant accuracy differences between wPLI and wSMI for each specific SNR that were observed for both intra- and inter-hemispheric conditions.

The topographic accuracy was defined as the proportion of simulated EEG datasets (with true interactions between the cortical sources of interest), in which the connectivity between the two electrodes closest to the cortical sources passed the 95th percentile of all other electrode pairings. Results are similar to those described for whole brain accuracy (Fig. 5). For the linear dynamics, wPLI and wSMI showed again similar mean accuracies, but wPLI tended to have higher accuracy for low SNRs (0.05–0.08) and high SNRs (>0.94). Accuracy of wPLI (but not of wSMI) further improved for band-limited connectivity in the alpha-range (8–12 Hz; Fig. S1), especially for low SNRs (0.04–0.09) as well as high SNRs (≥0.93). For both Hénon and Ikeda iterated maps the mean topographic accuracy of wPLI was significantly higher than the mean topographic accuracy of wSMI. Specifically, in the Hénon case wPLI had higher accuracy especially for SNRs ≥ 0.44, while in the Ikeda case it had higher accuracy at low and intermediate SNRs (0.14–0.50). Both wPLI and wSMI showed high levels of mean accuracy for the three Rössler cases (x, y; x, z; y, z), although wPLI performed significantly better than wSMI in the intra-hemispheric case of Rössler (x, y), the interhemispheric case of Rössler (x, z) and both inter-and intra-hemispheric cases of Rössler (y, z). The evaluation of accuracy levels as a function of SNR showed that wSMI tended to perform better than wPLI for low SNRs (0.11–0.21) in the Rössler (y, z) case, while it showed a steep decrease in accuracy at high SNRs (R-R Rössler (x, y) ≥0.75; L-R Rössler (x, z) ≥0.86; L-R/R-R Rössler (y, z) ≥0.76/0.83). Finally, while wPLI and wSMI showed similar mean accuracy in the Lorenz (x, y) case (with wPLI performing relatively better for SNRs in the range 0.03–0.06), only wSMI was able to detect interactions based on Lorenz (x, z) and Lorenz (y, z) dynamics (Lorenz (x, z) ≥0.07; Lorenz (y, z) ≥0.04).

 A video of the mean wPLI and wSMI connectivity matrices across all simulated hd-EEG recordings as a function of SNR for all nine investigated dynamics can be found in Supplementary Material Movie S1. Moreover,  a video of frequency-resolved wPLI computed between the electrodes spatially closest to the sources can be found in Supplementary Material Movie S2.

### Experimental data in wakefulness and sleep

**Figure 6:**
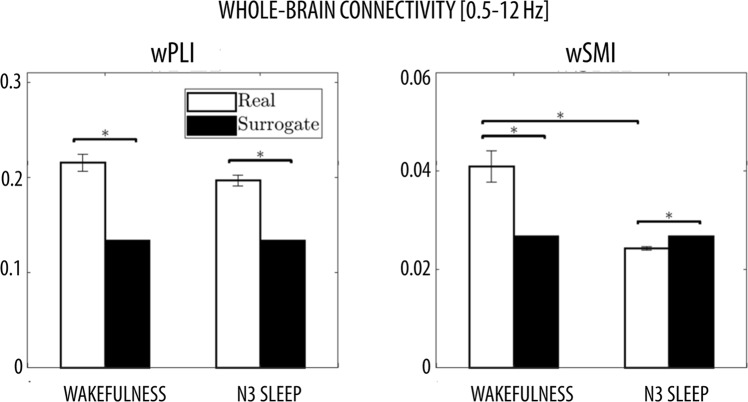
Whole-brain wPLI (left) and wSMI (right) connectivity in wakefulness and sleep (0.5–12 Hz). Paired comparisons were performed between median whole-brain connectivity in wakefulness and N3-sleep, as well as between experimental and surrogate data. * marks p < 0.05 (non-parametric permutation tests).

**Figure 7:**
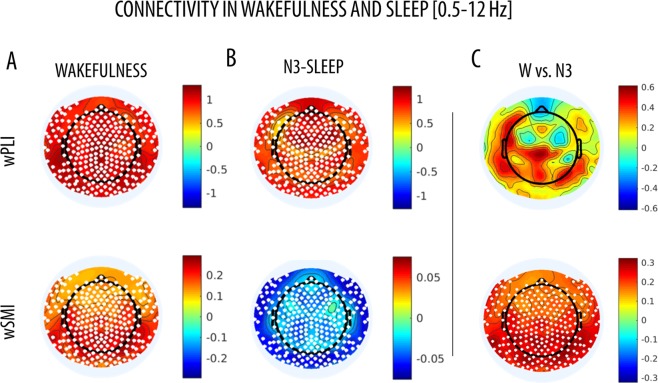
Topographic wPLI (left) and wSMI (right) connectivity in wakefulness and sleep (0.5–12 Hz). Paired comparisons were performed between (**A**) wakefulness and shuffled surrogate data and (**B**) N3-sleep and shuffled surrogate data and (**C**) wakefulness and N3-sleep, for wPLI (top row) and wSMI (bottom row). White dots mark significant effects (cluster-based non-parametric permutation test, p < 0.05). Colorbars show the permutation test statistic for the difference between conditions, so that the red color marks higher values in real vs. surrogate data for panels A and B. In panel C, the red color indicates higher values in wakefulness, while the blue color indicates higher values in sleep. These images were generated using the ‘topoplot’ function in EEGLAB^70^.

In wakefulness, both wPLI and wSMI revealed significant levels of connectivity in all tested electrodes (p < 0.05, cluster-corrected), relative to values observed in time-point shuffled data (Figs 6 and 7A; 0.5–12 Hz frequency range). In particular, for both measures the highest connectivity values were observed in posterior (occipital, parietal) areas. However, in N3-sleep the two methods provided different results: wPLI revealed diffuse high connectivity values peaking in frontal areas, while wSMI showed reduced connectivity values (Figs 6 and 7B). In line with these observations, the direct contrast between wakefulness and N3-sleep also revealed distinct changes based on wPLI and wSMI (Figs 6 and 7C). Specifically, while wSMI connectivity was significantly higher for wakefulness as compared to N3-sleep in all areas, there were no statistically significant differences in wPLI between these two states of vigilance.

**Figure 8:**
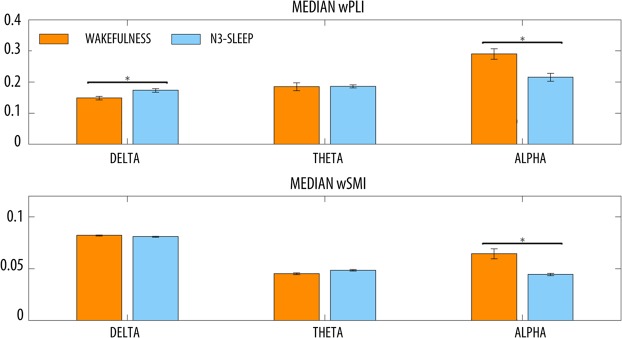
Whole-brain, median wPLI and wSMI in wakefulness (W) and N3-sleep in different frequency bands (delta: 0.5–4 Hz, theta: 4–8 Hz, alpha: 8–12 Hz). Error bars show the standard error of the mean. Horizontal bars and * mark significant differences between conditions (non-parametric permutation tests, p < 0.05, Bonferroni-corrected for the number of tested frequency bands).

**Figure 9:**
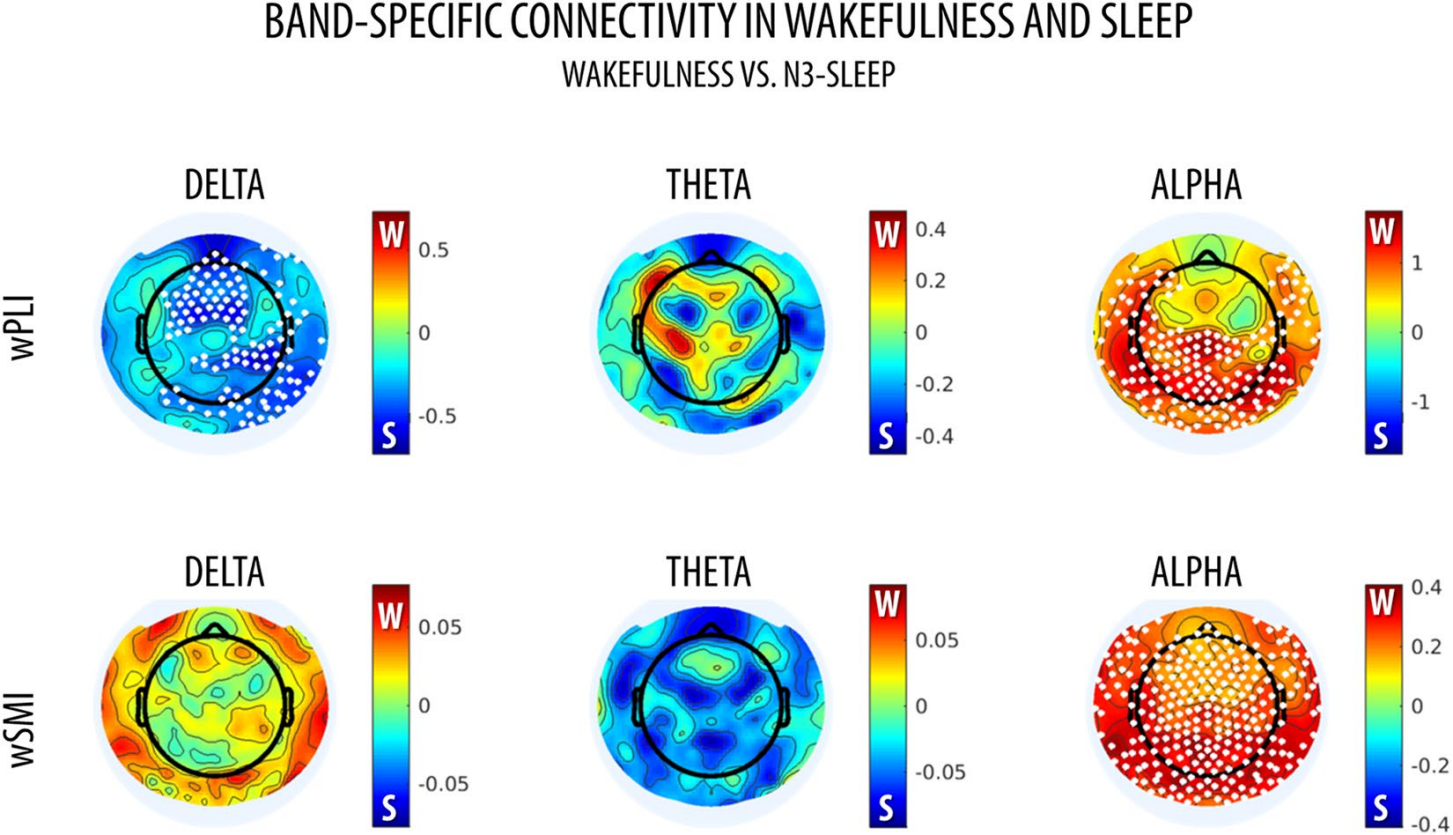
Topographic wPLI and wSMI connectivity in wakefulness (W) and N3-sleep in different frequency bands (delta: 0.5–4 Hz, theta: 4–8 Hz, alpha: 8–12 Hz). Colorbars show the permutation test statistic for the differences between wakefulness and N3-sleep. The red color marks higher values in wakefulness, while the blue color indicates higher values in sleep. White dots mark significant differences between conditions (cluster-based non-parametric permutation test, p < 0.05). These images were generated using the ‘topoplot’ function in EEGLAB^70^.

Further analyses focusing on classical frequency bands (delta: 0.5–4 Hz, theta: 4–8 Hz, alpha: 8–12 Hz), showed that both wPLI and wSMI were higher in wakefulness than in sleep within the alpha-band (Figs 8 and 9). However, wPLI was also lower in wakefulness relative to N3 in the delta-band. Frequency-resolved wPLI for wakefulness and sleep can be found in Supplementary Fig. S3.

## Discussion

The weighted Phase Lag Index^1^ and the weighted Symbolic Mutual Information^4^ are two robust functional connectivity approaches increasingly applied to M/EEG data, because of their relative immunity to volume conduction effects^5–19,25^. Here we set out to investigate whether the two methods are able to capture overlapping or complementary information regarding variations in brain inter-regional interactions. By combining analyses on simulated hd-EEG data and real hd-EEG recordings collected in different states of vigilance, we demonstrated that wPLI has an optimal sensitivity for interaction dynamics presenting a mixture of linear and nonlinear components, whereas wSMI has higher sensitivity to predominantly nonlinear dynamics. Given that the brain is a highly complex system typically characterised by both linear and nonlinear interaction dynamics^40^, it may be better described through the combined use of different measures^30^. Consistent with this view, our results suggest that the conjoint use of wPLI and wSMI may allow researchers to obtain complementary information about FC interactions, and thus to better describe relative changes associated with distinct behavioural states.

### Performance of wPLI and wSMI in simulated data

The ‘*Berlin Brain Connectivity Benchmark’* (BBCB) framework^33^ was adapted and employed to generate hd-EEG recordings in sensor-space. This framework allowed us to model different interaction dynamics between two cortical sources, noise with temporal and spatial structure as well as source mixing due to volume conduction, in a highly realistic electromagnetic volume conductor (head) model. We generated interaction dynamics with different degrees and types of nonlinearity, from linear to exclusively nonlinear, and specifically tested the sensitivity of wPLI and wSMI at detecting these inter-regional dependencies. For each of the considered dynamics, we also tested two different source locations (intra- and inter-hemispheric interactions) and different signal-to-noise ratios (SNR). Our results showed that the phase-based measure wPLI performs generally better at detecting inter-regional couplings presenting both linear and nonlinear components. Only in two of the more complex nonlinear coupling cases (Lorenz (x, z) and Lorenz (y, z)), characterised by non-significant cross-correlation values (see Figs 4 and 5), wPLI had a very low accuracy. Contrarily, the information-theoretic measure wSMI had a significantly higher accuracy for these two interaction dynamics, but performed significantly worse for the Ikeda-based couplings and also had lower topographic accuracy for Hénon- and Rössler-based couplings.

With few exceptions, the accuracies of wPLI and wSMI were very similar for intra- and inter-hemispheric interactions, and the detection accuracy of both methods tended to increase with an increase in SNR. Of note, the spatial (topographic) accuracy of wSMI (but not the whole-brain accuracy based on median global connectivity) showed instead a decrease at high SNRs for linear and Rössler interactions. This accuracy reduction may be related to an increase in the spatial spreading of the source signals to more distant scalp electrodes with increasing SNRs, which may have led a greater proportion of electrodes to detect the underlying functional coupling (loss of spatial resolution). Moreover, at high SNRs a relative ‘cross-contamination’ may be expected to occur between the two electrodes spatially closest to the interacting sources. In particular, the activity of one source may be ‘volume-conducted’ to the electrode closest to the other source (and vice-versa). Due to the particular weighting approach used for wSMI, the increased similarity between the signals of these particular channels may limit the maximum attainable connectivity strength, thus reducing the relative difference with respect to all other electrode pairings. On the other hand, such effects of volume conduction at high SNRs can be expected to have had only a marginal impact on (or even to improve) the estimation of whole-brain accuracy with respect to null-datasets generated from point- or phase-shuffled time-series.

In the linear case, where interacting dynamics were fixed in the alpha-range, we noted that both wPLI and wSMI had a lower accuracy at detecting the presence of interacting sources when evaluating the broadband signal instead of the band-limited one (Supplementary Fig. S1). As described below, this was confirmed by the analysis on experimental data, which revealed a higher sensitivity of the band-limited analysis to potential differences across vigilance states (Fig. 9). These observations indicate that wPLI and wSMI may have a lower sensitivity when computed on a frequency range larger than the one in which the interaction actually occurs. For this reason, a-priori knowledge regarding the potential frequency ranges of interest should be used to guide the analyses whenever possible. In this respect, wPLI has the important advantage of also allowing more exploratory, frequency-resolved analyses; however, such analyses may raise statistical issues when many distinct interactions have to be tested.

Overall, our results demonstrated that wPLI, as a measure of phase synchronization, performs generally better at detecting functional couplings presenting a mixture of linear and nonlinear dynamics, whereas wSMI, fundamentally rooted in information theory, has higher sensitivity for exclusively nonlinear dynamics, such as Lorenz (x, z) and Lorenz (y, z) dynamics. Importantly, present results also demonstrated that both wPLI and wSMI are characterised by a high spatial (topographic) accuracy, thus supporting their use in graph theoretical analysis at sensor-level.

### Performance of wPLI and wSMI in distinct states of vigilance

To evaluate whether the results we obtained from simulated EEG data are relevant to the analysis of real experimental data, we tested and compared the performance of the two connectivity measures in hd-EEG recordings collected in humans in different states of vigilance. In fact, both wPLI and wSMI have been previously shown to successfully identify relative variations in brain FC associated with different degrees of consciousness under anaesthesia or following severe brain injury^4,8–10,17,18^. Based on these premises, here we asked whether the two methods may identify similar or distinct changes associated with variations in the level of consciousness of healthy subjects from wakefulness to deep NREM-sleep (N3). In humans, N3-sleep is characterised by the occurrence of large and diffuse EEG slow waves (0.5–4 Hz), by relative sensory disconnection^41^ and by a low probability of having any conscious experiences (dreams)^42^. It has been suggested that slow waves, representing the alternation of neuronal silence (*off-period*) and firing (*on-period*), and occurring out-of-phase in different cortical areas, may contribute to the fading of consciousness through the interruption of causal interactions between distant brain regions^43–46^.

Here we showed that N3-sleep is associated with a significant and diffuse decrease in wSMI connectivity within the 0.5–12 Hz frequency range. This difference appeared particularly prominent in posterior brain areas. In contrast, we observed no significant differences between wakefulness and N3-sleep in broadband wPLI-connectivity. A band-limited analysis revealed that changes in wSMI were mainly driven by an overall decrease in *alpha* (8–12 Hz) connectivity in N3 relative to wakefulness. Of note, *alpha*-band wPLI connectivity also showed a similar, but more localized, decrease during N3-sleep, especially in posterior areas. These results are in line with previous work showing that the transition into unconsciousness due to sedation or physiological sleep (stage N1/N2) is associated with a decrease in alpha wPLI-connectivity^7,9,10,18,47^, especially in posterior regions^9,10^ and for posterior-anterior interactions^7,18,47^. Moreover, they are consistent with evidence indicating that relative to healthy individuals, patients with *unresponsive wakefulness syndrome* (UWS) or in a *minimally conscious state* (MCS) display a connectivity decrease that mainly affects posterior areas or posterior-anterior interactions^4,8,17,19,48^. Similarly, alpha-band wSMI has been found to be lower in UWS as compared to MCS patients^8^. Therefore, our findings indicate that both wPLI and wSMI may be suited to capture variations in *alpha*-connectivity associated with relative changes in vigilance and/or responsiveness to the environment. However, wPLI also revealed a relative increase in *delta* (0.5–4 Hz) connectivity. Importantly, the change in *delta*-wPLI is consistent with the presence of traveling slow waves during sleep^49^ as well as with a recent similar observation of increased parietal and parieto-frontal *delta*-wPLI connectivity during propofol sedation^9^ and midazolam-based anaesthesia^10^. Moreover, wPLI in the *delta*/*theta*-band has been shown to be increased in patients with disorders of consciousness (UWS, MCS), relative to healthy awake subjects^17^.

In summary, the analysis of wPLI- and wSMI-based connectivity in different states of vigilance confirmed our findings in simulated data, indicating that the two methods are sensitive to distinct brain dynamics. While an in-depth characterization of the differences in FC between wakefulness and sleep was beyond the scope of the present work, our results also suggest that wakefulness may be characterised by a mixture of ‘simple’ (i.e., mainly linear; better described by wPLI) and more complex (i.e., mainly nonlinear) interactions (better described by wSMI) in the alpha range, while sleep may be dominated by ‘simpler’ *delta*-band connectivity (better captured by wPLI), likely reflecting the occurrence of traveling slow waves. This interpretation is in line with previous observation indicating that N3 is associated with lower complexity or entropy^40,50^ as compared to wakefulness.

## Conclusions

Our study demonstrates that wPLI and wSMI connectivity metrics provide distinct but complementary information about inter-regional interactions and indicate that the combined use of these two methods may provide a better and more complete characterization of brain functional dynamics within and across distinct behavioural states. In particular, we showed that while wPLI displays an optimal sensitivity for interaction dynamics with linear and nonlinear components, wSMI has a higher sensitivity for predominantly nonlinear dynamics. We also showed that this finding may have significant implications for the analysis of functional connectivity in states of vigilance associated with different levels of consciousness. In light of recent evidence indicating that the independent application of wPLI and wSMI connectivity metrics may allow to identify changes in brain connectivity associated with variations in the level of consciousness, our results point to their possible combined use as a powerful tool to increase their accuracy and predictive value. Nonetheless, our findings may also have more general implications for the study of functional connectivity in a wide variety of behavioural conditions characterised by distinct underlying brain dynamics.

## Materials and Methods

### Ethics statement

The collection of experimental EEG data in wakefulness and sleep was approved by the ethical committee of the Canton of Vaud (Switzerland) and performed in accordance with relevant guidelines and regulations. Written informed consent was obtained from each subject.

### Simulation of hd-EEG data

The MATLAB-based ‘*Berlin Brain Connectivity Benchmark*’ (BBCB) framework^33^ was used to simulate realistic hd-EEG recordings (108 channels, 500 Hz, 120 s). In particular, the simulated electrical activity was generated by imposing bivariate relationships between two cortical sources, which were then projected at scalp level using a biophysically realistic model of electrical current propagation in the head. The adopted model was based on the standard ICBM152 anatomical template^51^ and included 6 tissue types: scalp, skull, air cavities, gray matter, white matter and cerebrospinal fluid (CSF). A finite element model (FEM) was solved to generate the lead field.

We modelled both intra- and inter-hemispheric interactions between pairs of cortical sources (see Fig. 1, including corresponding MNI coordinates). Specifically, the first source was placed either in left (LIPL) or right (RIPL) inferior parietal lobule, while the second source was kept in the right middle frontal gyrus (RMFG). The choice of these locations was motivated by previous neuroimaging studies showing that resting-state activity of these areas is modulated by conscious perception and attention^34–37^. Moreover, studies that employed wPLI and wSMI to investigate functional connectivity in different states of consciousness specifically suggested that a key correlate of such changes may be represented by variations in the strength of interactions across posterior and anterior brain areas^7,18,47^. For the sake of simplicity, only two interacting sources at a time were considered: LIPL-RMFG (inter-hemispheric) and RIPL-RMFG (intra-hemispheric).

As detailed below, we simulated nine different coupling relationships between the two sources, which differed in the type and relative degree of linear and nonlinear components. For each pair of source locations (LIPL-RMFG and RIPL-RMFG) and each type of simulated source coupling dynamics we also modelled 100 different signal-to-noise ratios (SNR; from 0.01 to 1, with steps of 0.01), which describe the weighting of simulated source signals with respect to simulated background activity. As detailed below, 100 different background noise patterns were obtained for each considered SNR. Specifically, brain noise ***n***_***b***_(*t*) was generated by placing 500 mutually statistically independent time-series characterised by 1/f-shaped power (pink noise) and random phase spectra at an equal number of random locations sampled from the entire cortical surface. Moreover, spatially and temporally uncorrelated sensor noise ***n***_***s***_(*t*) was sampled from a univariate standard normal distribution. The overall noise contribution was defined as noise n(t):

1 $$n(t)=0.9\frac{{n}_{b}(t)}{{\Vert {n}_{b}(t)\Vert }_{F}}+0.1\frac{{n}_{s}(t)}{{\Vert {n}_{s}(t)\Vert }_{F}},$$

where *n*(*t*)_*F*_ is the Frobenius norm. The simulated hd-EEG recording was generated according to:

2 $${x}^{int}(t)=\alpha \frac{{s}^{int}(t)}{{\Vert {s}^{int}(t)\Vert }_{F}}+(1-\alpha )\frac{n(t)}{{\Vert \tilde{n}(t)\Vert }_{F}},$$

where s^int^ corresponds to the signal contribution of the sources of interest to the EEG scalp signal (i.e. s^int^ is the projected source interaction to the EEG sensors through multiplication of the lead field with the source time courses, mapped to two patches of the cortical surface). The parameter α is related to the signal-to-noise and $\mathop{n}\limits^{ \sim }(t)$ is the filtered version of n(t) in the frequency-range of interest (8–12 Hz for linear dynamics, 0.5–12 Hz for the non-linear dynamics).

### Source interaction dynamics

For each source pairing (LIPL-RMFG, RIPL-RMFG), nine different coupling relationships were simulated by modelling the time-series of the two sources based on linear (AR) and nonlinear (Hénon^52^, Ikeda^53^, Rössler^54^, Lorenz^55^) dynamical systems.

#### Linear interactions

The time courses of the two sources were modelled using bivariate linear autoregressive (AR) models of order 5:

3 $$[\begin{array}{c}{z}_{1}(t)\\ {z}_{2}(t)\end{array}]=\sum _{p=1}^{P}[\begin{array}{cc}{a}_{11}(p) & {a}_{12}(p)\\ {a}_{21}(p) & {a}_{22}(p)\end{array}]\,[\begin{array}{c}{z}_{1}(t-p)\\ {z}_{2}(t-p)\end{array}]+[\begin{array}{c}{\varepsilon }_{1}(t)\\ {\varepsilon }_{2}(t)\end{array}]$$

where *a*_*ij*_(*p*), *i*,*j* ∈ {1, 2}, *p* ∈ {1, .., *P*} are linear AR coefficients, and ε_*i*_(t), *i* ∈ {1, 2} are uncorrelated standard normal distributed noise variables. The off-diagonal entry *a*_12_(*p*) was set to zero, while *a*_21_(*p*) was set to 0.5. Thus, interactions arise from a unidirectional time-delayed influence of z_1_ on z_2_. Moreover, the generated time series were bandpass-filtered in the alpha band (8–12 Hz) using an acausal third-order Butterworth filter with zero phase-delay^33^. We decided to simulate alpha oscillations with a clearly defined sender-receiver relationship, as they are also a key feature of brain activity in physiological wakefulness^56^.

#### Nonlinear interactions

Several distinct non-linear dynamics were selected in order to represent a wide-range of possible functional interactions. Among the chosen dynamics, the Hénon map and the Rössler systems have previously been employed by Wang *et al*.^57^ to test different functional connectivity measures. The time courses of the two sources were modelled by considering each one as a time-varying state variable of a specific dynamical system. In particular, we considered four different nonlinear systems: two defined by two-dimensional non-iterated maps (Hénon^52,57^ and Ikeda^53^) and two represented by three-dimensional nonlinear ordinary differential equation systems (Rössler^54,57^ and Lorenz^55^). Dynamical systems describe the motion of a point in a multidimensional state space, where the starting point is defined by the initial conditions of the system. For each system all potential combinations of variables have been considered as representing different interaction dynamics, i.e. Hénon (x, y), Ikeda (x, y), Rössler (x, y), Rössler (x, z), Rössler (y, z), Lorenz (x, y), Lorenz (x, z), Lorenz (y, z). The MATLAB-based Chaotic Systems Toolbox was used to compute the time series for the selected nonlinear systems, and the respective parameters were chosen to achieve complex chaotic behaviour: Hénon map [a = 1.4; b = 0.3], Ikeda map [μ = 0.9], Rössler dynamics [a = 0.2, b = 0.2, c = 5.7, *x*_0_ = 0.1, *y*_0_ = 0.1, *z*_0_ = 0.1, *h* = 0.1], Lorenz dynamics [σ = 10, β = 28, $\varrho$ = 8/3, *x*_0_ = 0.1, *y*_0_ = 0.1, *z*_0_ = 0.1, *h* = 0.1]. Due to the complex nature of these dynamics, they have not been limited to a specific frequency band.

### Connectivity analysis

The simulated EEG datasets (108 channels, 500 Hz, 120 s) generated for each coupling model were divided into 60 non-overlapping 2 s-epochs^1,4,58^. Then FC was computed for each epoch and pair of electrodes. While wPLI and wSMI could be theoretically applied to source-modelled EEG data, they are most commonly applied at scalp-level. For this reason all present analyses were performed by computing connectivity values between pairs of scalp EEG-sensors. Analyses were focused on the 0.5–12 Hz frequency range. Before computing connectivity measures, a current-source-density transform^59^ was applied to the EEG data, as in previous works^1,4^. This method provides a reference-independent signal and acts as a spatial filter, leading to a relatively improved spatial resolution^60^.

#### wPLI

The wPLI measures the extent to which phase angle differences between two time series *x*(*t*) and *y*(*t*) are distributed towards positive or negative parts of the imaginary axis in the complex plane (similar to the PLI^1,20^). The underlying idea is that volume-conducted activity accounts for the greatest proportion of detected 0° or 180° phase differences between signals. Therefore, to obtain a conservative estimate for real, non-volume conducted activity, only phase angle distributions predominantly on the positive or negative side are considered. The PLI is defined as the absolute value of the sum of the signs of the imaginary part of the complex cross-spectral density *S*_*xy*_ of two real-valued signals *x*(*t*) and *y*(*t*) at time point or trial *t*.

4 $${\rm{PLI}}=|\frac{{\sum }_{t=1}^{n}sgn(imag({S}_{xy,t}))}{n}|$$

While PLI is already insensitive to zero-lag interactions, the weighted Phase-Lag Index^1^ further addresses potential confounds caused by volume conduction, by scaling contributions of angle differences according to their distance from the real axis, as almost ‘almost-zero-lag’ interactions are considered as noise affecting real zero-lag interactions:

5 $${\rm{wPLI}}=|\frac{{\sum }_{t=1}^{n}|imag({S}_{xy,t})|sgn(imag({S}_{xy,t}))}{{\sum }_{t=1}^{n}|imag({S}_{xy,t})|}|$$

The wPLI is based only on the imaginary component of the cross-spectrum, and thus implies robustness to noise compared to coherence, as uncorrelated noise sources cause an increase in signal power^21^. Here wPLI was computed using the Fieldtrip toolbox^61^ (multi-taper method fast Fourier transform, single Hanning taper^1^, 0.5 Hz frequency resolution). The mean value across the frequency-bins in the frequency range of interest was computed to obtain a single wPLI coupling value (Broadband 0.5–12 Hz; Delta 0.5–4 Hz; Theta 4–8 Hz; Alpha 8–12 Hz).

#### wSMI

The wSMI^4^ evaluates the extent to which two EEG signals present non-random joint fluctuations, suggesting sharing of information. The time series *X* and *Y* in all EEG channels are first transformed into sequences of discrete symbols ($\hat{X}$, $\hat{Y}$). The symbols are coded according to the trends in amplitudes of a specific predefined number of consecutive time points. We chose the kernel *k* to be 3, implying that the symbols are constituted of three elements, leading to 3! = 6 different potential symbols in total^4,8^. The temporal separation of elements that constitute a symbol was set to be τ = 14 frames (***τ***_***t***_ = 28 *ms)*, such that the maximum resolved frequency was ${{f}}_{{\max }}=\frac{{fs}}{\mathrm{kx}{\tau }}=\frac{500{Hz}}{3{x}14}=11.9\,{Hz}$. Prior to wSMI computation, the signal was low-pass-filtered using the ‘*ft_preproc_lowpass*’ FieldTrip function with an additional mirror padding (‘*ft_preproc_padding*’) of 1 s before and after each individual epoch to avoid potential filter edge-artifacts. For the analysis in frequency bands, a bandpass-filter (‘*ft_preproc_bandpass*’) was used with the same padding scheme. For the additional computation of wSMI in delta- and theta- bands in experimental EEG recordings, τ was chosen accordingly (τ = 41 frames and τ = 21 frames, respectively). 

The joint probability of each pair of symbols co-occurring in two different time series is computed to estimate the symbolic mutual information (SMI) shared across two signals. To address volume conduction artifacts, the weighted symbolic mutual information disregards co-occurrences of identical or opposite-sign signals.

6 $${\rm{wSMI}}(\hat{X},\hat{Y})=\frac{1}{log(k!)}{\sum }_{\hat{x}{\epsilon }\hat{X}}{\sum }_{\hat{y}{\epsilon }\hat{Y}}w(\hat{x},\hat{y})p(\hat{x},\hat{y})log\frac{p(\hat{x},\hat{y})}{p(\hat{x})p(\hat{y})}$$

The wSMI can lead to negative values, given that it is a weighted mutual information measure, a form of weighted relative entropy^62^.

### Statistical procedure for simulated data

The accuracy of wPLI and wSMI was evaluated at *whole-brain* and *topographic* levels, respectively indicating *i*) the ability to detect the presence of statistical dependencies in the overall (median) connectivity across all pairs of electrodes (see Fig. 2), and *ii*) the ability to detect a significant interaction between the pairs of electrodes spatially closest to the actual brain sources among all pairs of electrodes.

#### Whole-brain accuracy

For each source pairing (LIPL-RMFG, RIPL-RMFG), tested interaction dynamics and SNR, the whole-brain detection accuracy of wPLI and wSMI was computed as the proportion of cases (N = 100 datasets differing by their respective spatial noise distributions), in which the whole-brain median connectivity value (across all electrode-pairs) of simulated EEG data passed the 95^th^ percentile of a null distribution obtained after time-point-shuffling of the original source-level timeseries (N = 100 permutations; Fig. 2). To account for the small number of permutations, a generalised Pareto distribution was used to model the tail of the null distribution, using the PALM (Permutation Analysis of Linear Models) software^63^. Of note, we chose to focus on a time-point-shuffling procedure instead of phase-shuffling, since the latter can introduce spurious interdependences between time-series, especially for the Rössler dynamics^64^. However, in the Supplementary Material, we also present results obtained with null distributions generated by phase-shuffling the original time-series using the Amplitude-Adjusted-Fourier-Transform (AAFT) procedure^65,66^. With the expected exception of the Rössler dynamics, the two approaches provided similar results (see Fig. S2).

#### Topographic accuracy

For each source location pairing, interaction dynamics and SNR, the topographic accuracy was defined as the proportion of simulated EEG datasets (N = 100 differing by their respective spatial noise distributions), in which the connectivity between the two electrodes spatially closest to the cortical sources (minimum Euclidean distance) passed the 95^th^ percentile of all other electrode pairings in each simulated EEG-recording with the same underlying brain noise (N = 5778 channel pairs).

In summary, for both approaches, a threshold corresponding to the 95^th^ percentile of the respective null-distributions (surrogate data for whole-brain connectivity, and connectivity of all electrode-pairs in topographic analysis) was regarded as the limit for the detection of significant FC interactions (α = 0.05). The mean total accuracy of wPLI and wSMI was computed as the mean of accuracies obtained across all SNRs. Non-parametric permutation tests (N = 10000, p < 0.05) were used to compare the performance of the two metrics at each SNR and for mean accuracy. Specifically, for each examined condition, the difference in mean accuracy between wPLI and wSMI was compared with a null distribution obtained by randomly ‘reassigning’ to the two metrics the values of accuracy determined for the different SNR configurations. A similar procedure was used to compare performance of wPLI and wSMI for different spatial distributions of noise at each SNR.

### Experimental hd-EEG recordings

To verify whether potential differences between wPLI- and wSMI-based FC metrics in recognising distinct interaction dynamics have actual implications for the analysis of real experimental data, an additional investigation was performed on hd-EEG recordings (257 channels, Electrical Geodeisics Inc.; 500 Hz) obtained in different behavioural states. Specifically, data were obtained from 12 healthy volunteers (25 ± 4 yrs, 6F) during distinct states of vigilance: relaxed wakefulness with eyes closed (W) and deep (N3-)sleep. The data was recorded as part of a larger project aimed at exploring the effects of changes in visual experiences during wakefulness on NREM-sleep features^67^.

Brain activity during N3-sleep was extracted from an overnight EEG recording in the sleep laboratory, whereas wakefulness data consisted of six minutes of eyes-closed resting-state activity obtained at 8AM the following morning, when homeostatic sleep pressure is expected to be at its minimum^68^. All continuous wake and N3-sleep recordings were band-pass filtered between 0.5 and 45 Hz (NetStation 5, EGI), and the first and last 5-s of data were discarded to account for filter-related edge-artifacts. Bad channels were identified upon visual inspection and interpolated using spherical splines: we removed 31.5 ± 12.9 electrodes (corresponding to 12.3 ± 5.0% of all electrodes) in sleep recordings, and 30.8 ± 8.6 electrodes (12.0 ± 3.4%) in wakefulness recordings. Sleep scoring was performed using standard procedures^69^ and all 30 s epochs containing N3-sleep were extracted and concatenated. EEG recordings during wakefulness were divided into non-overlapping 5 s segments and visually inspected to identify and reject clear artifacts. Overall, 27.3 ± 13.8% of all epochs were discarded due to artifacts, while in deep sleep no epochs were discarded. Indeed, large artifacts caused by eye movements, movements or muscular activity are typically absent or greatly reduced while in deep sleep.

For both wakefulness and sleep data, a procedure based on Independent Component Analysis (ICA) was used to remove residual ocular, muscular, and cardiac artifacts^70^. For each subject, we randomly extracted and analyzed the minimum common number (across subjects) of artifact-free 2s-long epochs of wakefulness data, corresponding to 70 segments (i.e. 140 s; the first 0.5 s and the last 0.5 s of each 5 s segment were discarded). The same amount of data (i.e. 70 2s-epochs; 140 s) was randomly selected from N3-sleep that occurred during the first half of the night. From this selection, we excluded epochs representing potential outliers in terms of signal power within classical frequency bands. Specifically, the Power Spectral Density (PSD; Welch’s method, Hamming windows, 8 sections, 50% overlap) of all N3 2s-epochs was calculated in delta (0.5–4 Hz), theta (4–8 Hz), alpha (8–12 Hz), sigma (12–16 Hz), beta (18–25 Hz), gamma (30–45 Hz) and broadband (0.5–45 Hz) frequency ranges. Then, outlier segments for any of the seven considered frequency ranges (i.e., threshold = median PSD ± 2 median absolute deviations; MAD) were excluded from the random selection procedure (see Fig. S4).

For each condition and channel, the median wPLI and wSMI connectivity of each electrode to all other scalp electrodes was computed in all epochs for the 0.5–12 Hz frequency range (i.e., as in simulated data). The median one-to-all connectivity of each electrode was computed and compared to the average of the median one-to-all connectivity across surrogate datasets (1000 iterations) generated through time-point shuffling of the original recordings of each channel. In this approach, the same permutation scheme was used for all subjects, and the global signal, corresponding to the average signal across all electrodes, was re-added to each shuffled dataset to ensure the preservation of the internal characteristics of the data and of the potential spurious (volume-conduction-dependent) interactions.

Paired comparisons were performed between i) wakefulness and surrogate data, ii) N3-sleep and surrogate data, and iii) wakefulness and N3-sleep (non-parametric permutation test; p < 0.05). Correction for multiple comparisons was ensured using a permutation-based supra-threshold cluster correction^71,72^. In brief, the same contrast was repeated (N = 10000 iterations) after shuffling the labels of the two compared sets and the maximum size of significant electrode-clusters was saved in a frequency table. A cluster-size threshold corresponding to the 95th percentile of the obtained distribution (α = 0.05) was applied to correct for multiple comparisons. Whole-brain connectivity (median of one-to-all connectivity across all electrodes) was also evaluated and compared to surrogate data using non-parametric permutation tests (N = 10000 iterations; p < 0.05).

## Supplementary information

Supplementary Material

Movie S1

Movie S2

## Data Availability

The scripts used for simultating and analysing the hd-EEG datasets are available from the first corresponding author on reasonable request. The ethical approval granted to the authors does not allow the publication of the raw EEG experimental data online. If readers would like to reanalyse the dataset, additional ethical approvals (on a individual user and purpose basis) will be required.

## References

[1] Vinck M, Oostenveld R, Van Wingerden M, Battaglia F, Pennartz CMA (2011). An improved index of phase-synchronization for electrophysiological data in the presence of volume-conduction, noise and sample-size bias. Neuroimage.

[2] Srinivasan R, Winter WR, Ding J, Nunez PL (2007). EEG and MEG coherence: Measures of functional connectivity at distinct spatial scales of neocortical dynamics. J. Neurosci. Methods.

[3] Khadem A, Hossein-Zadeh G-A (2014). Quantification of the effects of volume conduction on the EEG/MEG connectivity estimates: an index of sensitivity to brain interactions. Physiol. Meas..

[4] King JR (2013). Information sharing in the brain indexes consciousness in noncommunicative patients. Curr. Biol..

[5] Canales-Johnson, A. *et al*. Integration And Differentiation Of Neural Information Dissociate Between Conscious Percepts. *bioRxiv* 1–36, 10.13140/RG.2.2.34646.24647 (2017).

[6] Lau TM, Gwin JT, McDowell KG, Ferris DP (2012). Weighted phase lag index stability as an artifact resistant measure to detect cognitive EEG activity during locomotion. J. Neuroeng. Rehabil..

[7] Comsa IM, Bekinschtein TA, Chennu S (2017). Transient topographical dynamics of the electroencephalogram predict brain connectivity and behavioural responsiveness during drowsiness. bioRxiv.

[8] Sitt JD (2014). Large scale screening of neural signatures of consciousness in patients in a vegetative or minimally conscious state. Brain.

[9] Lee M (2017). Network Properties in Transitions of Consciousness during Propofol-induced Sedation. Sci. Rep..

[10] Lee, M. *et al*. Change in functional networks for transitions between states of consciousness during midazolam-induced sedation. In *Proceedings of the Annual International Conference of the IEEE Engineering in Medicine and Biology Society, EMBS* 958–961, 10.1109/EMBC.2017.8036984 (2017). 10.1109/EMBC.2017.8036984 29060032

[11] Simor, P., van der Wijk, G., Gombos, F. & Kovacs, I. Sharpening the paradox of REM sleep: cortical oscillations, synchronization and topographical aspects during phasic and tonic REM microstates. *J. Sleep Res*. **27** (2018).

[12] Ortiz, E. *et al*. Weighted phase lag index and graph analysis: Preliminary investigation of functional connectivity during resting state in children. *Comput. Math. Methods Med*. **2012** (2012). 10.1155/2012/186353 PMC3462418 23049617

[13] Parra, M. A. *et al*. Brain Information Sharing During Visual Short-Term Memory Binding Yields a Memory Biomarker for Familial Alzheimer’s Disease. *Curr. Alzheimer Res*. **14** (2017). 10.2174/1567205014666170614163316 28641509

[14] Tramonti C (2018). Predictive value of EEG connectivity measures for motor training outcome in multiple sclerosis: an observational longitudinal study. Eur. J. Phys. Rehabil. Med..

[15] Robinson SE, Mandell AJ (2015). Mutual information in a MEG complexity measure suggests regional hyper-connectivity in schizophrenic probands. Neuropsychopharmacology.

[16] Xing M (2017). Resting-state theta band connectivity and graph analysis in generalized social anxiety disorder. NeuroImage Clin..

[17] Chennu Srivas, Finoia Paola, Kamau Evelyn, Allanson Judith, Williams Guy B., Monti Martin M., Noreika Valdas, Arnatkeviciute Aurina, Canales-Johnson Andrés, Olivares Francisco, Cabezas-Soto Daniela, Menon David K., Pickard John D., Owen Adrian M., Bekinschtein Tristan A. (2014). Spectral Signatures of Reorganised Brain Networks in Disorders of Consciousness. PLoS Computational Biology.

[18] Chennu S, O’Connor S, Adapa R, Menon DK, Bekinschtein TA (2016). Brain Connectivity Dissociates Responsiveness from Drug Exposure during Propofol-Induced Transitions of Consciousness. PLoS Comput. Biol..

[19] Chennu S (2017). Brain networks predict metabolism, diagnosis and prognosis at the bedside in disorders of consciousness. Brain.

[20] Stam CJ, Nolte G, Daffertshofer A (2007). Phase lag index: Assessment of functional connectivity from multi channel EEG and MEG with diminished bias from common sources. Hum. Brain Mapp..

[21] Peraza LR, Asghar AUR, Green G, Halliday DM (2012). Volume conduction effects in brain network inference from electroencephalographic recordings using phase lag index. J. Neurosci. Methods.

[22] Schoffelen JM, Gross J (2009). Source connectivity analysis with MEG and EEG. Hum. Brain Mapp..

[23] Palva S, Palva JM (2012). Discovering oscillatory interaction networks with M/EEG: Challenges and breakthroughs. Trends Cogn. Sci..

[24] Hipp JF, Hawellek DJ, Corbetta M, Siegel M, Engel AK (2012). Large-scale cortical correlation structure of spontaneous oscillatory activity. Nat. Neurosci..

[25] Cohen MX (2015). Effects of time lag and frequency matching on phase-based connectivity. J. Neurosci. Methods.

[26] Gollo, L. L., Mirasso, C., Sporns, O. & Breakspear, M. Mechanisms of Zero-Lag Synchronization in Cortical Motifs. *PLoS Comput. Biol*. **10** (2014). 10.1371/journal.pcbi.1003548 PMC3998884 24763382

[27] Roelfsema PR, Engel AK, König P, Singer W (1997). Visuomotor integration is associated with zero time-lag synchronization among cortical areas. Nature.

[28] Casali, A. G. *et al*. A Theoretically Based Index of Consciousness Independent of Sensory Processing and Behavior. **5** (2013). 10.1126/scitranslmed.3006294 23946194

[29] West, T. *et al*. The Parkinsonian Subthalamic Network: Measures of Power, Linear, and Non-linear Synchronization and their Relationship to L-DOPA Treatment and OFF State Motor Severity. *Front. Hum. Neurosci*. **10** (2016). 10.3389/fnhum.2016.00517 PMC5078477 27826233

[30] David O, Cosmelli D, Friston KJ (2004). Evaluation of different measures of functional connectivity using a neural mass model. Neuroimage.

[31] Ince RAA (2016). A statistical framework for neuroimaging data analysis based on mutual information estimated via a Gaussian copula. Hum. Brain Mapp..

[32] Nir Yuval, Massimini Marcello, Boly Melanie, Tononi Giulio (2013). Sleep and Consciousness. Neuroimaging of Consciousness.

[33] Haufe, S. & Ewald, A. A Simulation Framework for Benchmarking EEG-Based Brain Connectivity Estimation Methodologies. *Brain Topogr*. 7562–7565, 10.1007/s10548-016-0498-y (2016).

[34] Vanhaudenhuyse A (2010). Default network connectivity reflects the level of consciousness in non-communicative brain-damaged patients. Brain.

[35] Vanhaudenhuyse A (2011). Two Distinct Neuronal Networks Mediate the Awareness of Environment and of Self. J. Cogn. Neurosci..

[36] Maksimow A (2005). Correlation of EEG spectral entropy with regional cerebral blood flow during sevoflurane and propofol anaesthesia. Anaesthesia.

[37] Martuzzi R, Ramani R, Qiu M, Rajeevan N, Constable RT (2010). Functional connectivity and alterations in baseline brain state in humans. Neuroimage.

[38] Quiroga, R. Q., Kraskov, A., Kreuz, T. & Grassberger, P. On the performance of different synchronization measures in real data: a case study on EEG signals. **65**, 1–14 (2001). 10.1103/PhysRevE.65.041903 12005869

[39] Arnhold J, Grassberger P, Lehnertz K, Elger CEE (1999). A Robust Method for Detecting Interdependences: Application to Intracranially Recorded EEG. Phys. D Nonlinear Phenom..

[40] Stam CJ (2005). Nonlinear dynamical analysis of EEG and MEG: Review of an emerging field. Clinical Neurophysiology.

[41] Rechtschaffen A, Hauri P, Zeitlin M (1966). Auditory awakening thresholds in REM and NREM sleep stages. Percept. Mot. Skills.

[42] Siclari F (2017). The neural correlates of dreaming. Nat. Neurosci..

[43] Massimini M (2005). Breakdown of cortical effective connectivity during sleep. Science.

[44] Jobst BM (2017). Increased Stability and Breakdown of Brain Effective Connectivity during Slow-Wave Sleep: Mechanistic Insights from Whole-Brain Computational Modelling. Sci. Rep..

[45] Vecchio F (2017). Cortical connectivity modulation during sleep onset: A study via graph theory on EEG data. Hum. Brain Mapp..

[46] Pigorini A (2015). Bistability breaks-off deterministic responses to intracortical stimulation during non-REM sleep. Neuroimage.

[47] Blain-Moraes S, Lee U, Ku S, Noh G, Mashour GA (2014). Electroencephalographic effects of ketamine on power, cross-frequency coupling, and connectivity in the alpha bandwidth. Front. Syst. Neurosci..

[48] Lehembre R (2012). Resting-state EEG study of comatose patients: a connectivity and frequency analysis to find differences between vegetative and minimally conscious states. Funct. Neurol..

[49] Massimini M, Huber R, Ferrarelli F, Hill S, Tononi G (2004). The Sleep Slow Oscillation as a Traveling Wave. J. Neurosci..

[50] Ma Y, Shi W, Peng CK, Yang AC (2018). Nonlinear dynamical analysis of sleep electroencephalography using fractal and entropy approaches. Sleep Medicine Reviews.

[51] Huang Y, Parra LC, Haufe S (2015). The New York Head-A precise standardized volume conductor model for EEG source localization and tES targeting. Neuroimage.

[52] Hénon M (1976). A two-dimensional mapping with a strange attractor. Commun. Math. Phys..

[53] Ikeda K (1979). Multiple-valued stationary state and its instability of the transmitted light by a ring cavity system. Opt. Commun..

[54] Rossler OE (1979). An equation for hyperchaos. Phys. Lett. A.

[55] Lorenz, E. The Lorenz System. 1–62 (1963).

[56] Sadaghiani S, Kleinschmidt A (2016). Brain Networks and α-Oscillations: Structural and Functional Foundations of Cognitive Control. Trends in Cognitive Sciences.

[57] Wang, H. E. *et al*. A systematic framework for functional connectivity measures. *Front. Neurosci*. **8** (2014). 10.3389/fnins.2014.00405 PMC4260483 25538556

[58] Colclough GLL (2016). How reliable are MEG resting-state connectivity metrics?. Neuroimage.

[59] Kayser J, Tenke CE (2006). Principal components analysis of Laplacian waveforms as a generic method for identifying ERP generator patterns: I. Evaluation with auditory oddball tasks. Clin. Neurophysiol..

[60] Nunez, P. L. & Srinivasan, R. Electric Fields of the Brain: The neurophysics of EEG. Electric Fields of the Brain: The neurophysics of EEG, 10.1093/acprof:oso/9780195050387.001.0001 (2009).

[61] Oostenveld R (2011). FieldTrip: Open Source Software for Advanced Analysis of MEG, EEG, and Invasive Electrophysiological Data, FieldTrip: Open Source Software for Advanced Analysis of MEG, EEG, and Invasive Electrophysiological Data. Comput. Intell. Neurosci..

[62] Kvålseth TO (1991). The relative useful information measure: Some comments. Inf. Sci. (Ny)..

[63] Winkler AM, Ridgway GR, Webster MA, Smith SM, Nichols TE (2014). Permutation inference for the general linear model. Neuroimage.

[64] Dahlhaus, R. *Mathematical methods in signal processing and digital image analysis*. (Springer, 2008).

[65] Theiler J, Eubank S, Longtin A, Galdrikian B, Doyne Farmer J (1992). Testing for nonlinearity in time series: the method of surrogate data. Phys. D Nonlinear Phenom..

[66] Theiler, J., Galdrikian, B., Longtin, A., Eubank, S. & Farmer, J. Using surrogate data to detect nonlinearity in time series. *Los Alamos Natl. Lab*. (1991).

[67] Bernardi Giulio, Betta Monica, Cataldi Jacinthe, Leo Andrea, Haba-Rubio José, Heinzer Raphaël, Cirelli Chiara, Tononi Giulio, Pietrini Pietro, Ricciardi Emiliano, Siclari Francesca (2019). Visual imagery and visual perception induce similar changes in occipital slow waves of sleep. Journal of Neurophysiology.

[68] Borbely AA (1982). A two process model of sleep regulation. Human Neurobiology.

[69] Iber, C., Ancoli-Israel, S. & A, C. The AASM manural for the scoring of sleep and associated events: Rules, terminology and technical specifications. *American Academy of Sleep Medicine* (2007).

[70] Delorme A, Makeig S (2004). EEGLAB: An open source toolbox for analysis of single-trial EEG dynamics including independent component analysis. J. Neurosci. Methods.

[71] Nichols TE, Holmes AP (2002). Nonparametric permutation tests for functional neuroimaging: A primer with examples. Hum. Brain Mapp..

[72] Huber R (2004). Local sleep and learning. Nature.

