# Supplementary material for: EEG functional connectivity metrics wPLI and wSMI account for distinct types of brain functional interactions
Source: Sci Rep. 2019 Jun 20;9:8894. doi: 10.1038/s41598-019-45289-7 (PMC6586889; doi:10.1038/s41598-019-45289-7)
Supplement: Supplementary file 1 — Supplementary Material [file 41598_2019_45289_MOESM1_ESM.pdf]

__Supplementary Material__

EEG functional connectivity metrics wPLI and wSMI account for distinct types of brain functional interactions

Laura Sophie Imperatori[1]*, Monica Betta[1], Luca Cecchetti[1], Andrés Canales-Johnson[2], Emiliano Ricciardi[1], Francesca Siclari[3], Pietro Pietrini[1], Srivas Chennu[4,5], Giulio Bernardi[1,3,6]*

[1] *MoMiLab Research Unit, IMT School for Advanced Studies Lucca, Lucca, Italy*
[2] *Department of Psychology, University of Cambridge, Cambridge, United Kingdom*
[3] *Center for Investigation and Research on Sleep, Lausanne University Hospital, Lausanne, Switzerland*
[4] *School of Computing, University of Kent, Chatham Maritime, United Kingdom*
[5] *Department of Clinical Neurosciences, University of Cambridge, Cambridge, United Kingdom*
[6] *University Hospital of Pisa, Pisa, Italy*

Figure S1

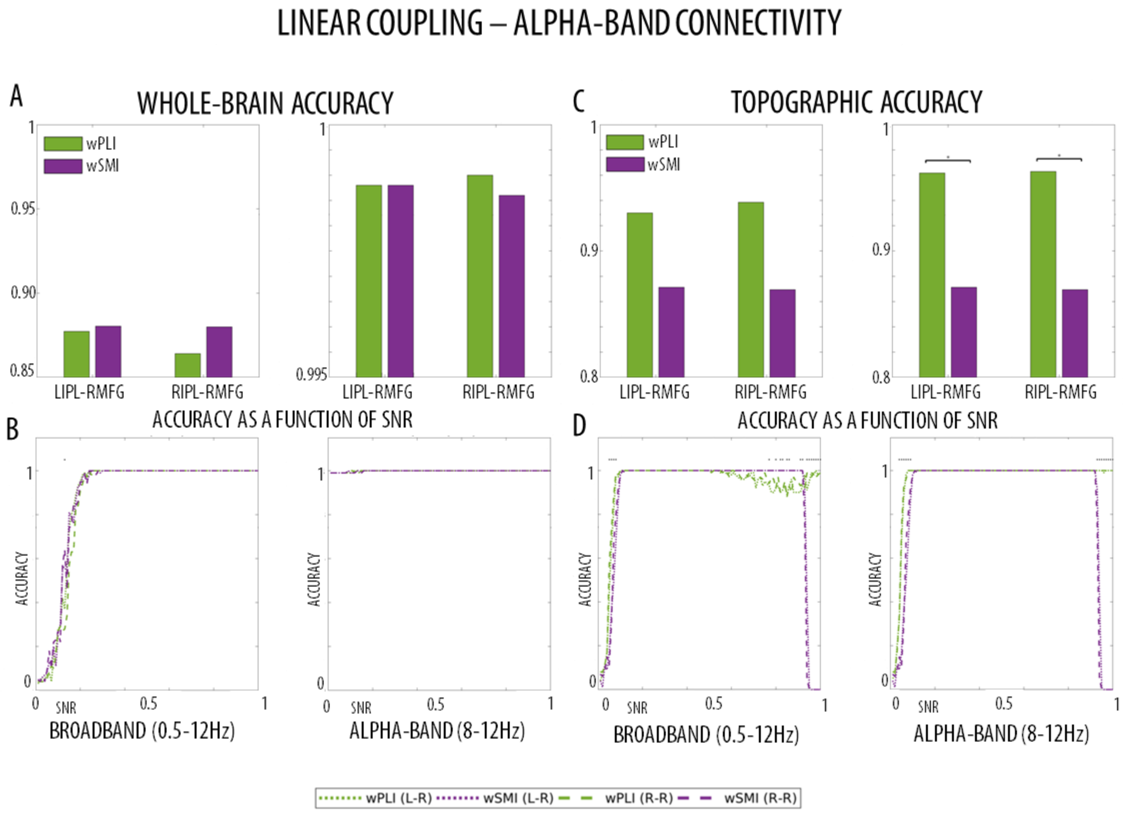

Figure S1. Whole-brain (A and B) and topographic (C and D) detection accuracy for linear dynamics analysed within the 0.5-12 Hz (broadband) or the 8-12 Hz (alpha-band) ranges (L = left IPL to right MFG; R = right IPL to right MFG). In bar plots (A, C), the * marks significant differences between wPLI and wSMI (permutation tests, $p < 0.05$). In B and D, accuracy is shown as a function of SNR. In these graphs, black dots mark significant accuracy differences between wPLI and wSMI (for a specific SNR) that were observed for both intra- and inter-hemispheric conditions.

Figure S2

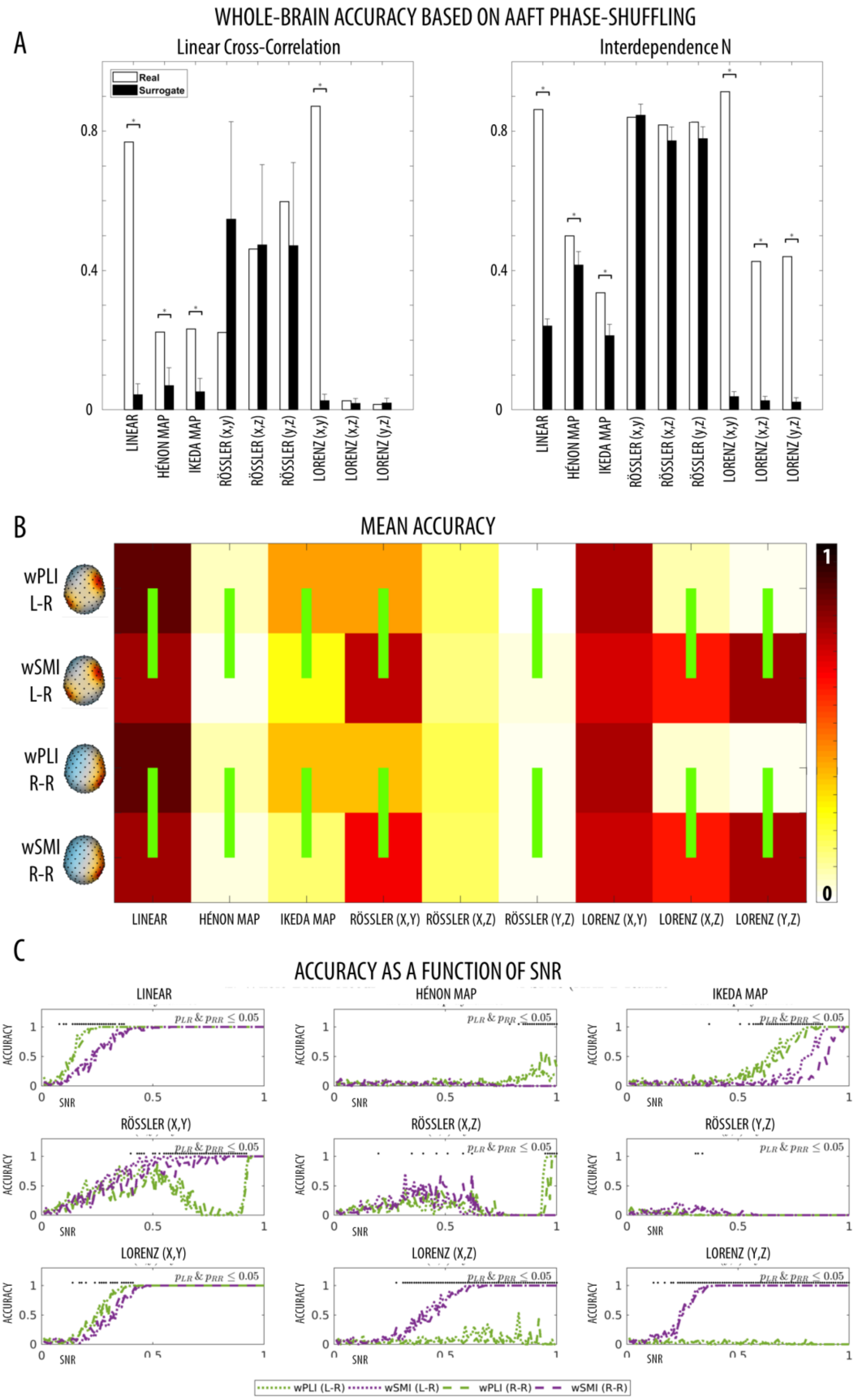

Figure S2. A) Absolute values of cross-correlation (CC; measure of similarity of two series as a function of the displacement of one relative to the other) and interdependence measure N (measure of the nonlinear relationship between two time series) for simulated source time-series (0.5-12 Hz) and for the corresponding time-series obtained after AAFT phase-shuffling (* for $p_{one\text{-}tail} < 0.05$, Bonferroni-corrected based on 18 comparisons). B) Mean whole-brain detection accuracy for the nine tested relationships between the chosen source location pairings (L = left IPL to right MFG; R = right IPL to right MFG). The green vertical lines mark significant differences between wPLI and wSMI (permutation tests, $p < 0.05$). C) Whole-brain detection accuracy for the nine tested relationships between the chosen source location pairings as a function of SNRs (L = left IPL to right MFG; R = right IPL to right MFG). Black dots at the top of each graph mark significant accuracy differences between wPLI and wSMI for each specific SNR that were observed for both intra- and inter-hemispheric conditions.

Figure S3

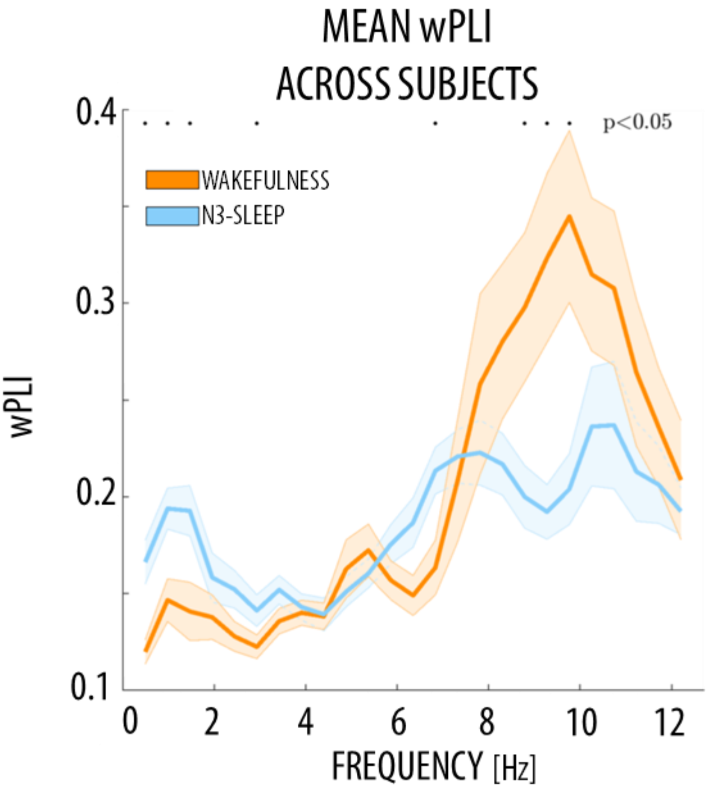

Figure S3. Group-level (N=12) average wPLI (median across channels) for each frequency-bin computed on data from wakefulness and N3-sleep. The shaded area reflects the standard error of the mean for each vigilance state. Black dots at the top mark significant differences between wakefulness and sleep for each specific frequency bin (non-parametric permutation tests, $p<0.05$).

Figure S4

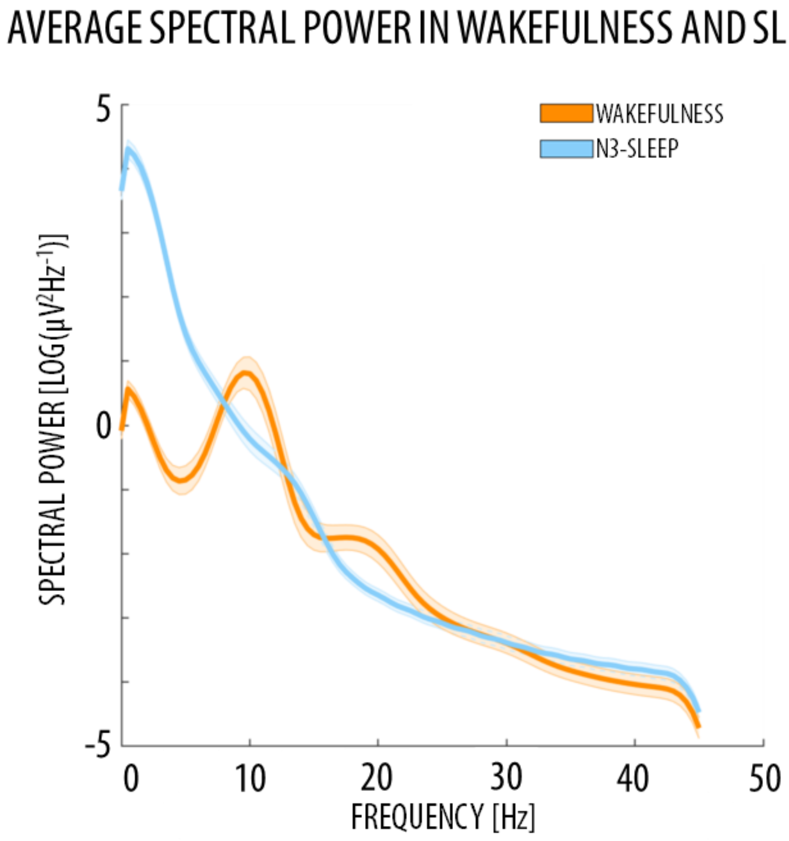

Figure S4. Group-level (N=12) average signal spectral power of data from wakefulness and N3-sleep. The shaded area reflects the standard error of the mean for each vigilance state.

Description of Supplementary Movies

Movie S1. Connectivity matrices obtained in the presence of interacting sources and in surrogate data as a function of SNR. Different videos have been generated for each of the tested interaction dynamics. The connectivity matrices represent the mean across all possible brain noise instantiations at any particular SNR. The top two connectivity matrices represent the matrices obtained in the presence of LIPL-RMFG or RIPL-RMFG interacting sources, while the bottom two connectivity matrices show the result of shuffled source interaction dynamics for both source location pairings. The top right plot shows the values used to determine topographic accuracy, i.e. the value of connectivity between the electrodes spatially closest to the sources, described here as "LIPL-RMFG" and "RIPL-RMFG" and their corresponding threshold, described as "95% All". The bottom right plot shows the values used to determine whole-brain accuracy, i.e. the value of median connectivity across all channels in real ("L-R Real" and "R-R Real") and surrogate data ("L-R Surr", "R-R Surr").

Movie S2. Movies of frequency-resolved wPLI computed between electrodes spatially closest to the sources at each SNR. The shaded area reflects the standard error of the mean for each source location pairing.
